# *De Novo* Transcriptome Sequencing of *Serangium japonicum* (Coleoptera: Coccinellidae) and Application of Two Assembled Unigenes

**DOI:** 10.1534/g3.119.400785

**Published:** 2019-11-12

**Authors:** Ya Hui Hu, Yong Liu, Lin Wei, Hao Tao Chen

**Affiliations:** Plant Protection Institute, Hunan Academy of Agricultural Science, Changsha, P.R. China, 410125

**Keywords:** *Serangium japonicum*, RNA sequencing, de novo assembly, overwinter, ladybird beetle

## Abstract

The ladybird beetle *Serangium japonicum* is an important predator of whiteflies. Investigations of the molecular mechanisms of this predatory beetle have been hindered by the scarcity of gene sequence data. To obtain gene sequences for the ladybird beetle and determine differences in gene expression between the summer and winter seasons, paired-end sequencing was performed. Real-time PCR was used to validate differences in Krueppel homolog 1 gene (*Kr-h1*) mRNA expression in summer *vs.* winter samples. To determined the diversity of the population, annotated cytochrome c oxidase subunit I gene (*COX1*) gene fragments were amplified from several ladybird beetle populations. The analysis yielded 191,246 assembled unigenes, 127,016 of which (66.4%) were annotated. These functional annotations of gene sequences are currently available from the National Center for Biotechnology Information (NCBI), and will provide a basis for studying the molecular mechanisms underlying the biological characteristics of *S. japonicum*. We found a change in expression of ribosome-associated genes across seasons, and postulate that this change is because of seasonal variation in temperature and photoperiod. The differential expression of *Kr-h1* suggests that *S. japonicum* can successfully overwinter because the adults enter diapause. To explain the effects of season on *Kr-h1* gene expression, we hypothesize a model in which that a short photoperiod affects the density of Ca^2+^, the subsequent activity of methyl farnesoate epoxidase and the synthesis of JH, and in turn *Kr-h1* gene expression. *COX1* annotation was concordant with the morphological ID. The same *COX1* sequence was found in the samples from several provinces in China. Therefore, the *COX1* sequence is worth further study to distinguish beetle species and populations.

Whiteflies are notable pests that prey on many horticultural crops (Ren *et al.* 2001; Ren *et al.* 2011). *Serangium japonicum* have been reported as an effective or potential predator of several types of whiteflies, such as *Bemisia tabaci* (Ren *et al.* 2001; Sahar and Ren 2004; Li *et al.* 2015), *Dialeurodes citri* (Kaneko 2017), and *Aleurocanthus camelliae* (Ozawa and Uchiyama 2016). *S. japonicum* has been studied for its biological characteristics (Yao *et al.* 2005; Fatiha *et al.* 2008; Yao *et al.* 2011; Li *et al.* 2014; Hu *et al.* 2016; Kaneko 2017) and response to insecticides (Hu *et al.* 2009; He *et al.* 2012; Zhao *et al.* 2012; Yao *et al.* 2015) and juvenile hormone analog (Li *et al.* 2015). Gene sequences from *S. japonicum* have not been reported by others.

Next-generation RNA sequencing has been used to assess the molecular mechanisms underlying processes in insects (Zhang *et al.* 2014; Qi *et al.* 2015). Compared with other molecular technologies, next-generation RNA sequencing has a lower cost. Transcriptome sequencing can effectively identify molecular markers (Parra-González *et al.* 2012). The assembled genes from the transcriptome can be expressed differently in selected tissues or organs, and at various developmental stages (Fu *et al.* 2016). Moreover, when a reference genome is unavailable, next-generation RNA sequencing can effectively obtain the annotated gene sequences of a species (Martin and Wang 2011) via BLAST (basic local alignment search tool) with other species’ gene sequences in several public databases.

Diapause is a behavior that allows insects to adapt to an unfavorable environment. Cold dormancy temperatures (5–8°) lasting for 30 days can be fatal to *S. japonicum* adults if diapause is not induced before they are exposed to such temperatures (Hu *et al.* 2016). Insects in diapause can survive in cold weather for several months, particularly if they have accumulated fatty material in the body before overwintering (Hahn and Denlinger 2011). Juvenile hormone (JH) can control developmental transitions in insects, including diapause. As transcription factor at downstream of *JH* signaling pathway (Jindra *et al.* 2013; Jindra *et al.* 2015), the *Krueppel homolog 1* (*Kr-h1*) gene plays an important role in inducing diapause. This project aimed to explore whether there is a difference of *Kr-h1* expression in *S. japonicum* in summer *vs.* winter.

The *cytochrome c oxidase subunit I* (*COX1*) gene can be used to identify different biological species (Hebert *et al.* 2004). In insects, *COX1* has been successfully used to distinguish between different whitefly species or biotypes and different geographical populations within a whitefly species (Ren *et al.* 2011). *S. japonicum* are difficult to distinguish from *Delphastus catalinae* because they have similar morphological features. Additionally, *S. japonicum* is distributed throughout many provinces in China and several other countries. It is expected that a *COX1* sequence fragment can be regarded as an effective DNA barcode of *S. japonicum*.

The purpose of this study was to provide abundant gene sequences for future studies of *S. japonicum*, find differences in gene expression between two seasons to identify potential reasons underlying differential gene expression, to focus on *Kr-h1* gene expression to search for the molecular mechanism underlying diapause in *S. japonicum*, and to evaluate the *COX1* sequence as a potential target for distinguishing species and populations.

## Materials and methods

### Species identification

The samples were identified as *S. japonicum* by vice professor Wang Xing-ming of South China Agriculture University. We also judged the species by referencing Jing *et al.* (2003). The adults were 1.8-2.0 mm × 1.4-1.5 mm, back and compound eye black, head, prosternum, and leg yellow, antenna like knife. They preyed on whitefly eggs and larvae.

### RNA extraction and sequencing

*S. japonicum* samples were collected from eggplants at the Academy of Hunan Agricultural Science, Changsha, China 3 times during winter 2016 and 3 times during summer 2017. Each time, 7 *S. japonicum* adults were collected from plants as a biological sample, for a total of 42 adults. Total RNA was extracted from *S. japonicum* with the EasyPure RNA kit (Transgen Biotech, Beijing, China). RNA was sequenced by Sangon Biotech (Shanghai) Co., Ltd., Shanghai, China, on an Illumina HiSeq 2500.

### De novo transcriptome assembly

Raw reads from 6 biological samples were cleaned by removing adapter sequences (forward: AGATCGGAAGAGCACACGTCTGAAC; reverse: AGATCGGAAGAGCGTCGTGTAGGGA) and removing bases from both sides that had Q < 20, removing reads with unknown nucleotides “N”, and removing reads <35 nucleotides in length. The clean reads from both groups were assembled *de novo* using Trinity (Haas *et al.* 2013). The Trinity default parameter setting was used, except for min_kmer_cov. Trinity treated the cleaned reads via 3 steps: Inchworm, Chrysalis, and Butterfly. None of assembled sequences <200 nucleotides were regarded as unigenes.

### Functional annotation

The generated unigenes were annotated based on the following 5 databases: the National Center for Biotechnology Information (NCBI) non-redundant protein database (Nr), the NCBI non-redundant nucleotide database (Nt), Swiss-Prot, the Eukaryotic Orthologous Groups (KOG) database, and the Kyoto encyclopedia of genes and genomes (KEGG), with E-value <1 × 10^−5^. The best-aligned results were used to decide the sequence direction and coding sequence of unigenes. If results of different databases conflicted with each other, the following order of priority was employed: Nr, Nt, Swiss-Prot, KEGG, and COG. Even if unigenes were not annotated in any of listed databases, their sequence direction and coding sequence would be predicted by TransDecoder (v3.0.1) (http://transdecoder.github.io/). Distribution of similar species was analyzed based on Nr database annotation (Shi *et al.* 2011).

### Kr-h1 expression validation in summer and winter

One microgram of RNA was employed for first-strand cDNA synthesis with a RevertAid Premium Reverse Transcriptase kit (Thermo Scientific) used according to the manufacturer’s instructions. Real-time polymerase chain reaction (PCR) was performed with SG Fast qPCR Master Mix (High Rox) at 95° for 3 min, followed by 45 cycles of 95° for 7 s and 56° for 10 s. The primers used were 5′-3′sequence TCAGGAACGCAGTTCTAC and 5′-3′ sequence AGTTAGGCGAGCAGGTACGG. Melting curves were analyzed from 60° to 95° to detect nonspecific product amplification. The assembled gene_id: TRINITY_DN79847_c1_g1 of *S. japonicum* was used as an internal control. Data analysis was carried out by the 2^-ΔΔCT^ method.

### COX1 gene as barcode investigation of the geographic populations

The investigated populations of *S. japonicum* were distributed in Changsha (northern latitude 28°, eastern longitude 113°) in Hunan province, Mianyang (northern latitude 31°, eastern longitude 104°) in Sichuan province, and Nanjing (northern latitude 32°, eastern longitude 119°) in Jiangsu province. 10 individuals per population were sequenced, with 1 individual per DNA sample. DNA was extracted with the EasyPure DNA kit (Transgen Biotech, Beijing, China). According to the transcriptome sequencing, assembled unigenes, and annotation from Nr, we designed the following primer pair: 5′-3′: TATTTTCTTTTTGGACTTTG, 5′-3′: GTAATGTTGCTAATCAAGAAAA. These primers amplified a 980-nucleotide *COX1* gene fragment from 3 populations via PCR. Sequences from each of the 3 *S. japonicum* populations were aligned.

### Statistical analysis

The level of unigene expression was estimated by measuring transcripts per million reads (TPM) (Patro *et al.* 2017). In addition, the DESeq test was used to identify differentially expressed genes between the respective TPMs of summer *vs.* winter samples, with *P* ≤ 0.05 and ≥twofold change. The p-value was corrected by multiple tests (Storey and Tibshirani 2003), with q-value ≤ 0.05 (File S3). The differentially expressed genes were examined by functional and pathway enrichment analysis using GO data and KEGG terms. For GO enrichment analysis, we chose the topGO 2.24.0 R Package. For KEGG enrichment analysis, we chose the clusterProfiler 3.0.5 R Package.

### Data availability

The raw reads produced in this study can be obtained in NCBI by searching the project numbers PRJNA376265 (https://www.ncbi.nlm.nih.gov/bioproject/PRJNA376265) and PRJNA430037 (https://www.ncbi.nlm.nih.gov/bioproject/PRJNA430037); BioSample numbers SAMN06347100, SAMN08365344, and SAMN08365343; or with the accession codes SRR5277648, SRR6473305, SRR6473306, SRR6473307, SRR6473308, and SRR6473309. The assembled unigene sequences have been submitted to the Transcriptome Shotgun Assembly sequence database with the accession code GGMU00000000.

File S1: Some common statistical results for the transcriptome sequences of six *S. japonicum* samples. File S2: GO-classed unigenes with differential expression.

(https://zenodo.org/record/3351768#.XTqta9IwjUI)

File S3: The different expression unigenes between summer and winter.

TPM: transcripts per million reads. pValue: statistical test p-value. qValue: corrected p-value after multiple tests.

(https://zenodo.org/record/3445935#.XYM5Q9IwjUI)

## Results

### Sequencing and assembly

**Figure 1:**
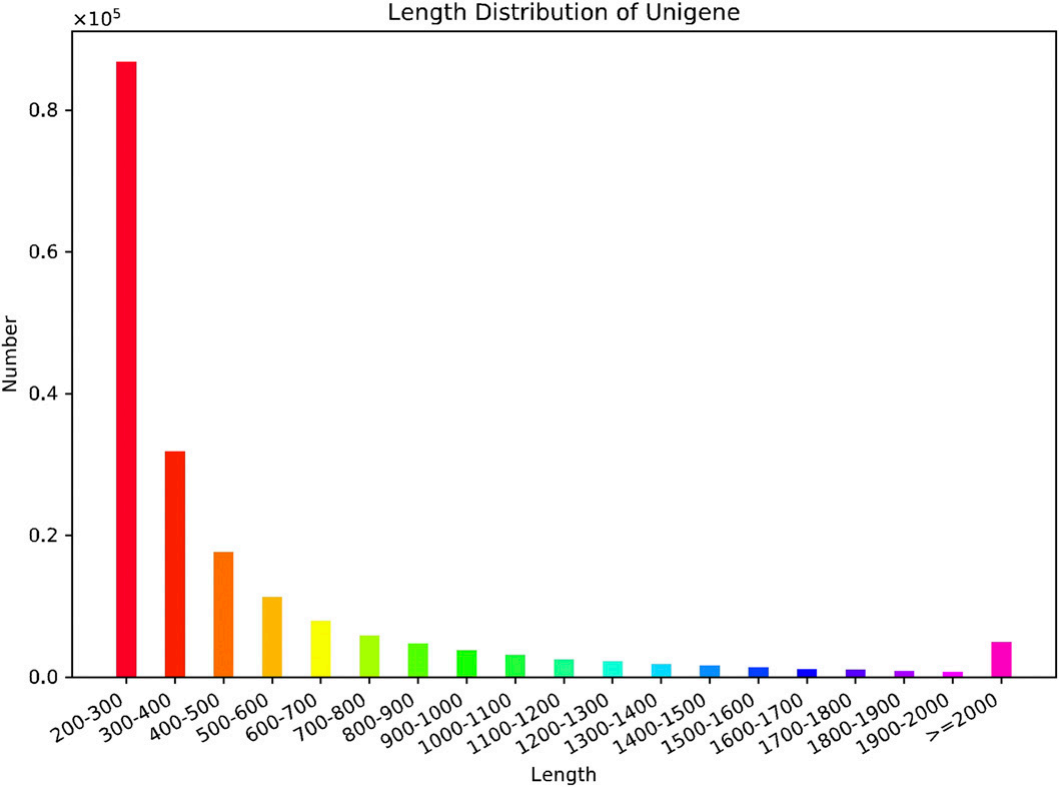
Length distribution of the obtained unigenes The x-axis represents the length, and the y-axis represents the number of unigenes.

More than 142 million raw reads were obtained from each group of samples (Table 1), resulting in 133.29 million cleaned reads (92.4% of the raw reads) for the 3 summer adult samples, and 140.34 million (93.7% of the raw reads) for the 3 winter adult samples. File S1 includes more detailed results of the raw and cleaned reads from six biological samples. Analysis yielded 191,246 unigenes with an average length of 524 nt. Half of these unigenes were longer than 681 nt, whereas 54,929 (28.7%) and 21,351 (11.2%) unigenes were longer than 500 nt and 1000 nt, respectively (Figure 1).

**Table 1:** Sequencing data from *S. japonica* samples collected in summer *vs.* winter

Total clean reads, N	133285022	140344314
Total clean bases, nucleotides	19145670934	19706386331
Q20 percentage of total reads	98.96%	98.64%
GC percentage of total nucleotides	44.19%	44.03%

### Functional annotation

Unigenes were annotated according to the Nr, Nt, Swiss-Prot, COG, KEGG database. After searches in these 5 databases, a total of 127,016 (66.4% of 191,246) unigenes could be annotated: 89,349 in Nr, 85,085 in Swiss-Prot, 79,088 in Nt, 62,877 in the KOG database, and 10,554 in KEGG.

**Figure 2:**
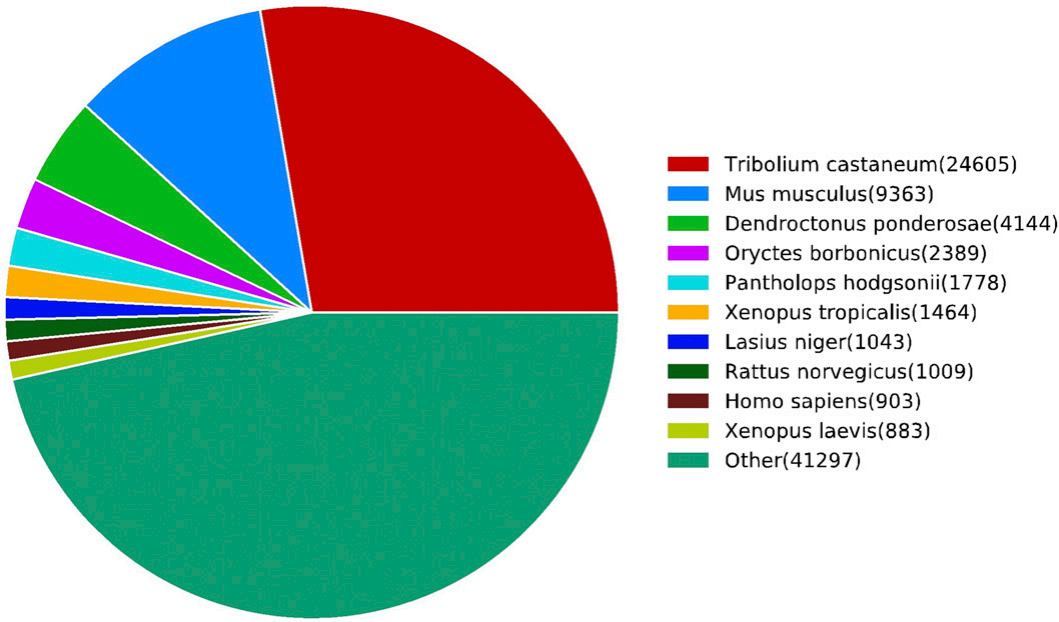
Distribution of species in the NCBI non-redundant protein database The species were determined based on the highest score of all of 191, 246 unigenes in the BLASTX results.

**Figure 3:**
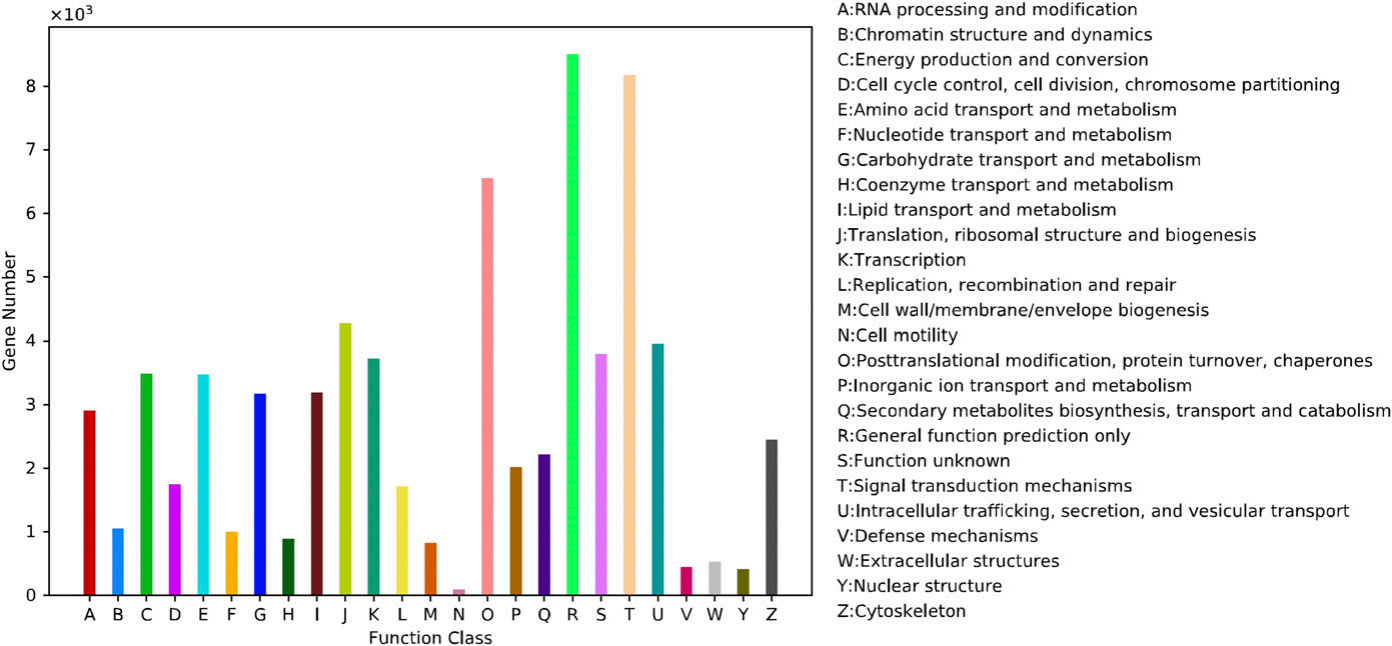
Eukaryotic orthologous group (KOG) categories A total of 62,877 unigenes were annotated into 25 categories. The y-axis represented number of unigenes.

According to the Nr database annotation, genes from *S. japonicum* were most similar to *Tribolium castaneum* (Figure 2). The RNA samples had not been contaminated by the presence of other species, for example, parasites. According to search results from the KOG database, the 3 largest categories were general function prediction (8503; 13.52%); signal transduction mechanisms (8175; 13.00%); and posttranslational modification protein turnover chaperones (6548; 10.41%) (Figure 3). According to the KEGG, a total of 24,653 unigenes were identified by 309 KEGG pathways; the most represented were ribosomal pathways (847 unigenes, 3.4%), followed by oxidative phosphorylation (502; 2.0%), carbon metabolism (431; 1.7%), and biosynthesis of amino acids (414; 1.7%). These functional annotations of unigenes provide a basis for studying the molecular mechanisms underlying the biological characteristics of *S. japonicum*.

### Different expression profiles in summer and winter

**Figure 4:**
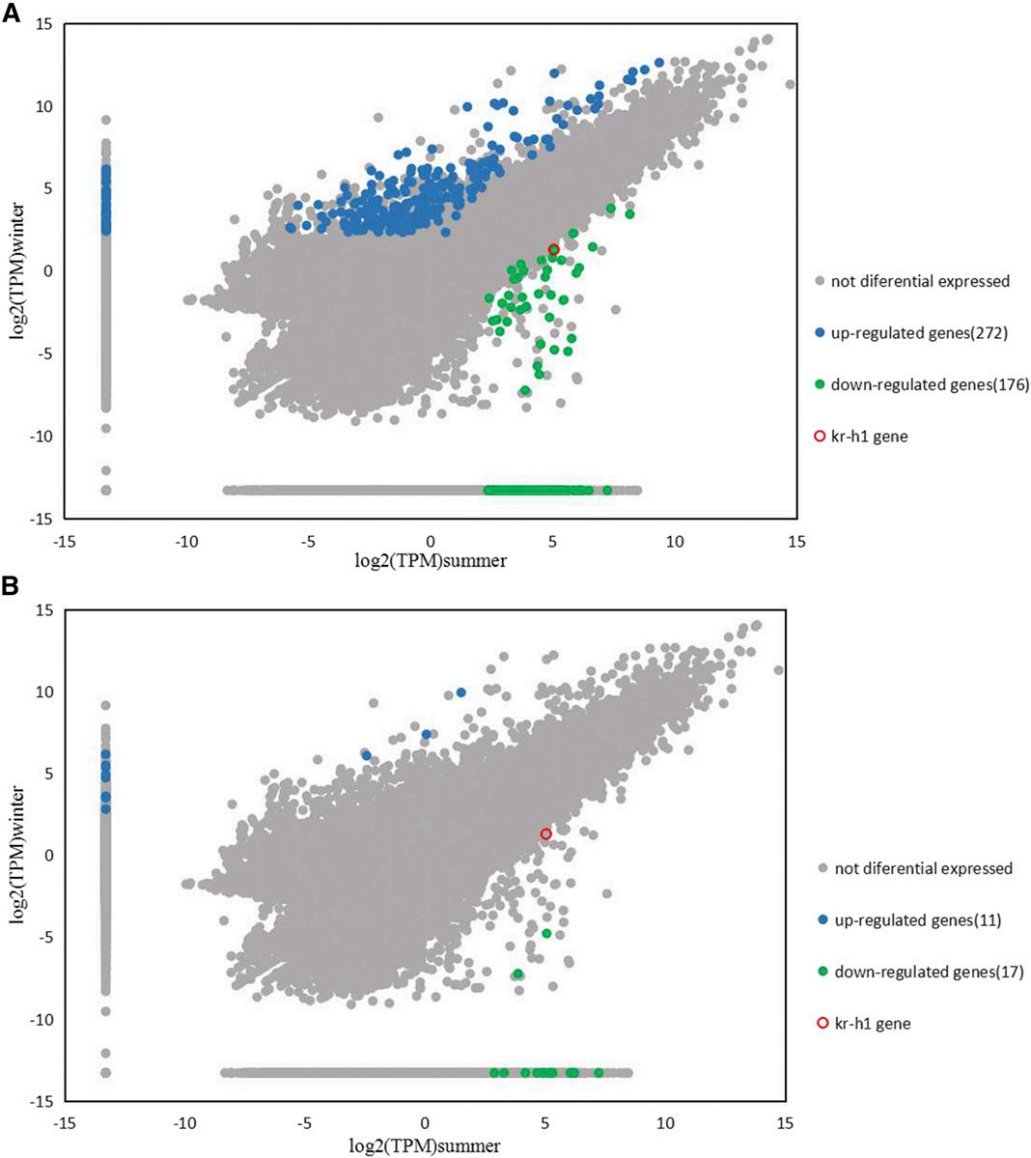
Expression profile of unigenes in two groups The x-axis represents log2(TPM)summer. The y-axis represents log2(TPM)winter. (A) Differentially expressed genes with *P* ≤ 0.05 before multiple tests. (B) Differentially expressed genes with corrected *P* ≤ 0.05 after multiple tests.

There were 448 unigenes differently expressed between the winter and summer samples, with 272 up-regulated and 176 down-regulated unigenes in winter (Figure 4A; File S3), when filtering differential unigenes with *P* ≤ 0.05 and ≥twofold change. Using the corrected p-value via multiple tests instead of the p-value, there were only 28 differentially expressed genes between winter and summer, with 11 up-regulated and 17 down-regulated unigenes in winter (Figure 4B; File S3). The number of up-regulated unigenes was less than the number of down-regulated unigenes after multiple test-correcting the p-value.

**Figure 5:**
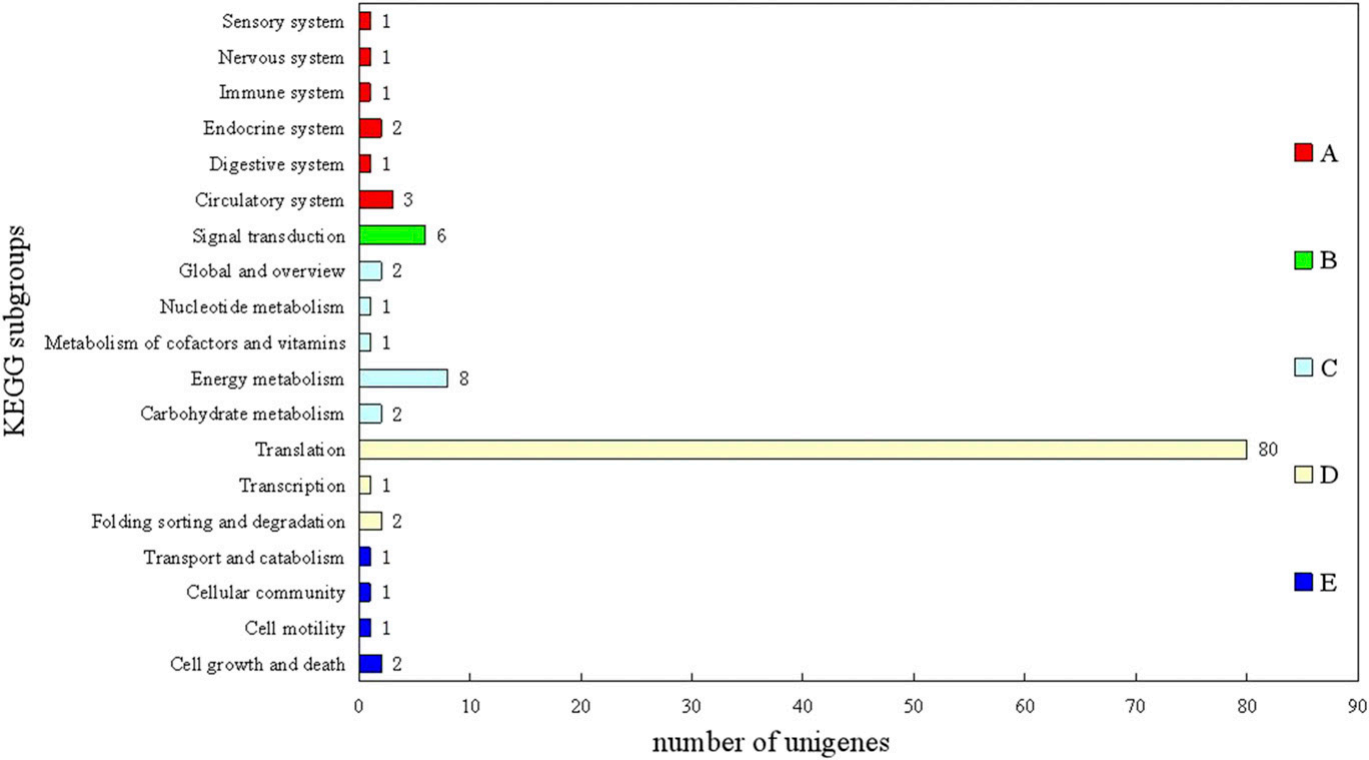
KEGG Orthology classifications of differentiallly expressed genes 117 KEGG annotated of 448 differential genes were classed into five groups: A: Organismal Systems; B: Environmental Information Processing; C: Metabolism; D: Genetic Information Processing; E: Cellular Processes. The y-axis represented subgroups. The x-axis represented number of unigenes.

448 differential genes were enriched significantly into 350 GO terms, with 275 biological process terms, 41 cellular component terms, and 34 molecular function terms (File S2). The most significantly enriched GO terms were ribosome, cytosolic ribosome, ribosomal subunit, cytosolic part, intracellular ribonucleoprotein complex, ribonucleoprotein complex, cytosolic large ribosomal subunit, and large ribosomal subunit. A total of 448 differential genes were classified into five groups and 19 subgroups based on the KEGG terms (Figure 5). The ribosome (KEGG id: ko03010) was unique significant enrichment pathway via analysis of differentially expressed genes between summer and winter.

### Kr-h1 expression in summer and winter

Expression of the *Kr-h1* gene (Gene id: TRINITY_DN74054_c1_g1) was down-regulated (Figure 4A; File S3) in *S. japonicum* in winter compared with summer (mean TPM values: 2.5 in winter *vs.* 33.1 in summer; log2Foldchange 3.75). The p-value was corrected to 0.518 from 0.038 via multiple testing (File S3). The q-PCR results for the *Kr-h1* gene showed log2Foldchange = 3.02 and *P* = 0.021.

### Application of the COX1 gene

The assembled sequence (Gene id: TRINITY_DN81324_c0_g4) was annotated as the gene for *COX1* according to the Nr, Nt, Swiss-Prot, and KOG databases. The PCR results showed the sequences were identical in *S. japonicum* in Changsha, Mianyang, and Nanjing according to a 928-nucleotide *COX1* fragment. In the NCBI nucleotide database, the assembled sequence had the highest identity value (91%) with the sequence ID KP829591.1 from *Serangium sp*. (Table 2).

**Table 2:** Taxonomy BLAST reports from the National Center for Biotechnology Information (NCBI) database (The unigene annotated as COX1 was used to BLASTN on the NCBI website at 07:37 January 28, 2019, with Query ID: lcl|Query 11215. Organisms were ranked according to number of hits.)

Organism	BLAST Name	Score	Number of Hits
Holometabola	insects		104
Coleoptera	beetles		90
Polyphaga	beetles		87
Cucujiformia	beetles		57
Cucujoidae	beetles		21
Coccinellidae	beetles		10
Serangium sp. CO631	beetles	1628	1
Scymninae sp. 2ACP-2013	beetles	1567	1
Propylea japonica	beetles	1506	1

## Discussion

### Season and gene expression

The ribosome pathway was significantly enriched by the differentially expressed unigenes in winter and summer. Much of the protein synthesis was affected among season, and more than one environmental factor changed with season. Most days *S. japonicum* lived at >26° and <10° in summer and winter, respectively. The photoperiod was longer than 12 h and shorter than 12 h in summer and winter, respectively. The number of up-regulated unigenes was less than the number of down-regulated unigenes when filtering the differentially expressed genes with a multiple test-corrected *P* ≤ 0.05. We concluded that many genes were expressed unstably among different samples in the same season due to several environmental factors.

### Kr-h1, diapause, and overwintering

Temperatures of 5–8° for 30 days can be fatal to *S. japonicum* adults if they do not enter diapause before exposure to the cold; in contrast, adults in diapause can survive for several months (Hu *et al.* 2016). JH acts together with the Methoprene-tolerant and Germ cell-expressed bHLH-PAS transcription factors (which act as potential JH receptors) to directly induce *Kr-h1* expression (Minakuchi *et al.* 2008; Lozano and Belles 2011; Kayukawa *et al.* 2012). Levels of the JH esterase and JH were low in diapause adults compared with non-diapause adults (Qi *et al.* 2015). The down-regulation of *Kr-h1* expression suggested that *S. japonicum* adults in winter were in diapause, with low JH levels. After diapause, the insect can successfully overwinter in low-temperature conditions because of the accumulation of fatty acids, trehalose, and other energy sources (Hahn and Denlinger 2011; Tang *et al.* 2017).

The *Kr-h1* gene negatively regulates ecdysone biosynthesis by directly inhibiting the transcription of steroidogenic enzymes (Liu *et al.* 2018; Zhang *et al.* 2018). A hormone receptor also acts as a repressor of ecdysone biosynthesis in *Drosophila melanogaster* (King-Jones *et al.* 2005; Ou *et al.* 2011). Other hormone receptors may inhibit ecdysone biosynthesis in *S. japonicum* adults. Future studies will explore whether ecdysone biosynthesis is inhibited during pre-diapause by high *Kr-h1* expression in *S. japonicum*. The relationship between *Kr-h1* and other hormone receptors in insects is also of increasing interest.

### Molecular mechanism of season affecting Kr-h1 gene expression

**Figure 6:**
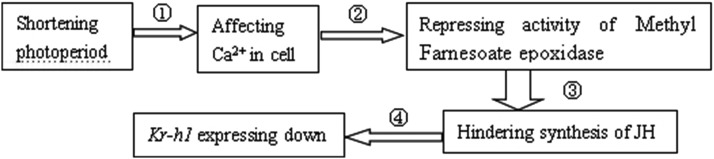
Molecular mechanism of photoperiod affecting *Kr-h1* gene expression Supporting data: ①: KEGG pathway id: ko04745 from fly; ②–③ Huang *et al.* 1994 from *Gryllus bimaculatus*; ④: Jindra *et al.* 2013 from many insects; Jindra *et al.* 2015 from many insects.

Diapause can be induced in *S. japonicum* by a shorter photoperiod (Hu *et al.* 2016). In this study, we found that the *Kr-h1* gene is down-regulated in winter. Thus, the *Kr-h1* gene was down-regulated more likely because of a shorter photoperiod rather than a lower temperature in winter. The titer of JH in *S. japonicum* was lower during diapause than non-diapause because *Kr-h1* is a JH transcription factor (Minakuchi *et al.* 2008; Lozano and Belles 2011; Kayukawa *et al.* 2012). Because of light affecting the density of Ca^2+^ in cells (KEGG pathway id: ko04745), and the density of Ca^2+^ affecting the activity of methyl farnesoate epoxidase (Huang *et al.* 1994), the mechanism underlying the effect of photoperiod on *Kr-h1* gene expression can be hypothesized (Figure 6): a short photoperiod affects the density of Ca^2+^, which affects the activity of methyl farnesoate epoxidase, which then affects the synthesis of JH, which finally affects *Kr-h1* gene expression.

Of course, it is also worth noting that other factors (like nutritional shifts due to availability of prey) than temperature and photoperiod could differ between seasons and could also be in play in season affecting *Kr-h1* gene expression.

### S. japonicum species and population

*Serangium sp*., *Scymninae sp*., and *Propylea japonica* have high *COX1* gene identity with *S. japonicum*. The COX1 from *S. japonicum* is concordant with the morphological ID, but more samples of *S. japonicum* should be used to test the accuracy of *COX1* before it has a DNA barcode of *S. japonicum*. The same *COX1* sequence was present in several provinces in China. We suggest that *S. japonicum* can be widely applied to control whitefly populations in many regions after the predator population expands/propagates. Further study is needed to determine the relationship between the *COX1* sequence and biological population of *S. japonicum*. Furthermore, as there are multiple populations of whitefly (Ren *et al.* 2011; Kanmiya *et al.* 2011), *S. japonicum* may have multiple populations if more samples from more regions of China were sequenced for *COX1*.

### Conclusions

*S. japonicum* is an effective predator of whiteflies. However, better use of this species requires thorough study of the molecular mechanisms underlying diapause, overwintering, and other biological characteristics. The study of molecular mechanisms of this predatory beetle is hindered by the scarcity of gene sequence data. The Illumina Hiseq2500 sequencing platform was used to sequence the *S. japonicum* transcriptome, yielding 191,246 assembled unigenes, of which 127,016 (66.4%) were annotated. This study identified an abundance of genes in *S. japonicum*. Annotation of unigenes would facilitate understanding of the mechanisms underlying biological characteristics in this species. The differential expression of ribosome relative genes showed that the synthesis of many proteins was affected by season. Many genes were expressed unstably among different samples in the same season due to several environmental factors. The seasonal differential expression of *Kr-h1* suggests that *S. japonicum* can successfully overwinter because the adults enter diapause. We hypothesize that the shorter photoperiod can result in *Kr-h1* down-regulation via the reduced density of Ca^2+^ affecting the activity of methyl farnesoate epoxidase. The *COX1* sequence is worthy of further study to distinguish beetle species and biological populations.
